# Caffeine intake antagonizes salt sensitive hypertension through improvement of renal sodium handling

**DOI:** 10.1038/srep25746

**Published:** 2016-05-12

**Authors:** Hao Yu, Tao Yang, Peng Gao, Xing Wei, Hexuan Zhang, Shiqiang Xiong, Zongshi Lu, Li Li, Xiao Wei, Jing Chen, Yu Zhao, William J. Arendshorst, Qianhui Shang, Daoyan Liu, Zhiming Zhu

**Affiliations:** 1 Center for Hypertension and Metabolic Diseases, Department of Hypertension and Endocrinology, Daping Hospital, Third Military Medical University, Chongqing Institute of Hypertension, Chongqing 400042, China; 2 Department of Cardiology, Institute of Clinical Medicine of Zunyi Medical College, Affiliated Hospital of Zunyi Medical College, Zunyi, Guizhou 563003, China; 3 Department of Cell Biology and Physiology, 6341-B Medical Biomolecular Research Building, CB #7545, School of Medicine, University of North Carolina at Chapel Hill, Chapel Hill, NC 27599-7545, USA.

## Abstract

High salt intake is a major risk factor for hypertension. Although acute caffeine intake produces moderate diuresis and natriuresis, caffeine increases the blood pressure (BP) through activating sympathetic activity. However, the long-term effects of caffeine on urinary sodium excretion and blood pressure are rarely investigated. Here, we investigated whether chronic caffeine administration antagonizes salt sensitive hypertension by promoting urinary sodium excretion. Dahl salt-sensitive (Dahl-S) rats were fed with high salt diet with or without 0.1% caffeine in drinking water for 15 days. The BP, heart rate and locomotor activity of rats was analyzed and urinary sodium excretion was determined. The renal epithelial Na^+^ channel (ENaC) expression and function were measured by *in vivo* and *in vitro* experiments. Chronic consumption of caffeine attenuates hypertension induced by high salt without affecting sympathetic nerve activity in Dahl-S rats. The renal α-ENaC expression and ENaC activity of rats decreased after chronic caffeine administration. Caffeine increased phosphorylation of AMPK and decrease α-ENaC expression in cortical collecting duct cells. Inhibiting AMPK abolished the effect of caffeine on α-ENaC. Chronic caffeine intake prevented the development of salt-sensitive hypertension through promoting urinary sodium excretion, which was associated with activation of renal AMPK and inhibition of renal tubular ENaC.

High salt intake is a major risk factor of hypertension^1^,^2^. Epidemiological studies demonstrate that more than 60% of hypertensive individuals are salt-sensitive^3^. Although reducing daily salt intake 2.0 to 2.3 grams can decrease deleterious cardiovascular events by 20%^4^, it is difficult to achieve this goal by health education. A survey showed that there was no significant reduction in salt intake in the US over the past 20 years^5^, and only a few countries meet the daily salt intake level of 6 g/day recommended by WHO^5^. Currently, several diuretic and natriuretic drugs are commonly used to promote the urinary sodium excretion in hypertensive patients^6^. Thus, it is crucial to find a more acceptable approach for promoting urinary excretion in normal or pre-hypertensive population.

Caffeine (1, 3, 7-trimethylxanthine) is the most commonly used psychoactive agent consumed daily by nearly 80% of the world’s population and 90% of adults in North America in forms of coffee, tea and food^7^,^8^. Caffeine was used historically to increase urine output before its more potent pharmacological diuretics became widely available^9^. Although acute effect of caffeine increases blood pressure and heart rate^10^,^11^, chronic caffeine administration prevents diet-induced insulin resistance^12^,^13^, attenuates metabolic syndrome through weight loss and reduction in fat mass^14^ as well as reduces fructose-induced hypertension^15^.

It is well documented that impaired renal sodium excretion plays a critical role in the pathogenesis of hypertension^16^,^17^. The thiazide-sensitive NaCl cotransporter (NCC), amiloride-sensitive epithelial sodium channel (ENaC), and sodium hydrogen (Na/H) exchanger are major regulators of renal tubular sodium reabsorption and of blood pressure^18^,^19^. Among them, ENaC mediates apical entry of sodium in the aldosterone-sensitive distal tubule and collecting duct and accounts for the rate-limiting step for sodium reabsorption in these nephron segments^20^. ENaC activity is stimulated by kinases such as SGK1 and PKA and inhibited by PKC, ERK1/2 and AMPK^21^. Caffeine can activate AMPK in different type of cells, including the vascular endothelial cells, skeletal muscle cells and hepatocytes^22^,^23^,^24^. However, it is unknown whether caffeine regulates ENaC function in distal nephron through AMPK or other kinases.

In the present study, we hypothesized that long-term caffeine consumption inhibits ENaC function in distal nephron, which in turn increases urinary sodium excretion and prevents hypertension induced by high salt intake. To test this hypothesis, the Dahl’s salt sensitive rat, a salt-sensitive hypertension model^25^,^26^, was examined in this study. We provide *in vivo* and *in vitro* evidence that AMPK activation by caffeine promoted urinary sodium excretion and lowered blood pressure through inhibition of the ENaC function in salt-sensitive rats. The present results also suggest that the acute and chronic actions of caffeine are mediated via different sites of the kidney tubules.

## Results

### Effect of chronic caffeine intake on blood pressure

**Figure 1:**
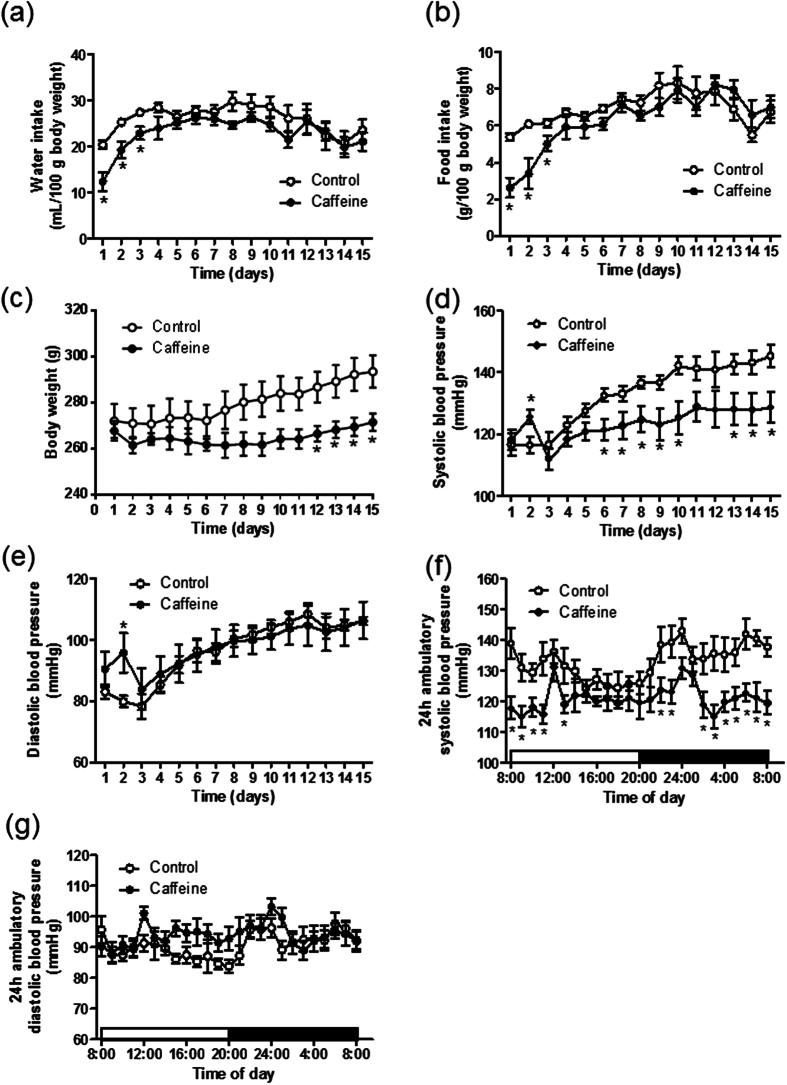
Chronic caffeine intake attenuates high salt induced hypertension. (**a**–**c**) The water, food intake and body weight analyses after high salt (control) or high salt plus caffeine (caffeine) intervention (n = 8). (**d**,**e**) Systolic blood pressure and diastolic blood pressure analyses in control or caffeine groups (n = 9). (**f**,**g**) 24 hr systolic and diastolic blood pressures in conscious Dahl-S rats on the 15^th^ day of caffeine intervention (n = 9). All data are presented as means ± SEM. *P < 0.05 compared with control.

Despite lower water and food intake in the first three days, chronic caffeine intake had no effect on water and food intake in Dahl-S rats thereafter (Fig. 1a,b), but significantly decreased their body weight compared with controls from the 12th day of treatment (Fig. 1c). Similarly, although caffeine intake temporally increased blood pressure on the second day, long-term caffeine intake attenuated high salt-induced increase in systolic, but not diastolic blood pressure in a time dependent manner (Fig. 1d,e). Also, 24-hr ambulatory systolic blood pressure on the 15th day was lower in caffeine-treated rats. This anti-hypertensive effect of chronic caffeine was more obvious at night when the rats were more active (Fig. 1f). 24 hr ambulatory diastolic blood pressure did not differ between the two groups (Fig. 1g).

### Effect of chronic caffeine intake on sympathetic nerve activity and vascular function

**Figure 2:**
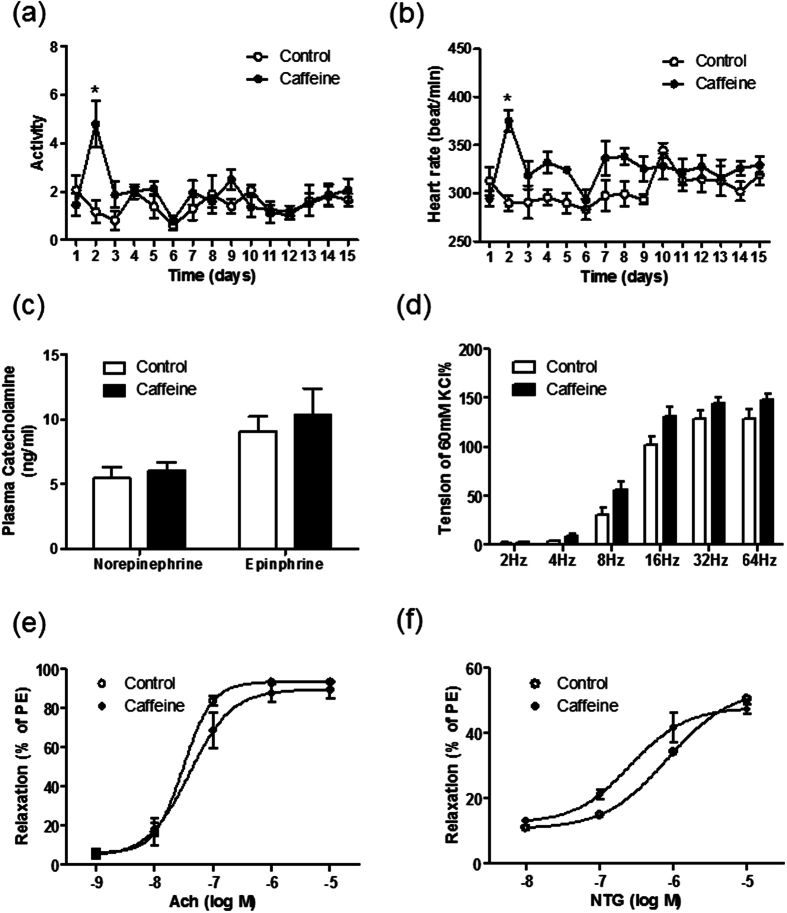
Chronic caffeine intake has no significant impact on sympathetic nerve system and cardiovascular system. (**a**,**b**) Locomotor activity and heart rate changes in control and caffeine groups (n = 5). (**c**) Plasma catecholamine concentration on the 16^th^ day (n = 6). (**d**) Electrical field stimulation-induced mesenteric arterial contraction is similar in control and caffeine groups. Results are expressed as a percentage of the initial contraction elicited by 60 mM KCl (n = 5). (**e**,**f**) Endothelium-dependent vasodilation induced by acetylcholine (Ach) was not significantly different in control and caffeine group (n = 5). Endothelium-independent vasodilation induced by nitroglycerin (NTG) was also similar in control and caffeine group (n = 5). Results are expressed as a percentage of the maximum contraction elicited by 10^−5^ M phenylephrine (PE). All data are presented as means ± SEM. *P < 0.05 compared with control.

On the second day, caffeine initially increased heart rate and locomotor activity, after that, there were no significant difference in heart rate and locomotor activity between control and caffeine-treated Dahl-S rats (Fig. 2a,b). Accordingly, the plasma catecholamine concentration was not different between the two groups (Fig. 2c). In addition to sympathetic nerve activity, electrical field stimulation (EFS)-induced mesenteric artery constriction was not affected by caffeine (Fig. 2d). Similarly, both of endothelium-dependent and independent relaxations of mesenteric arteries were almost equal between the two groups (Fig. 2e,f). These results indicate that neither sympathetic nerve activity nor vascular function is responsible for the anti-hypertensive effect of chronic caffeine intake.

### Chronic caffeine intake increases urinary sodium excretion

**Figure 3:**
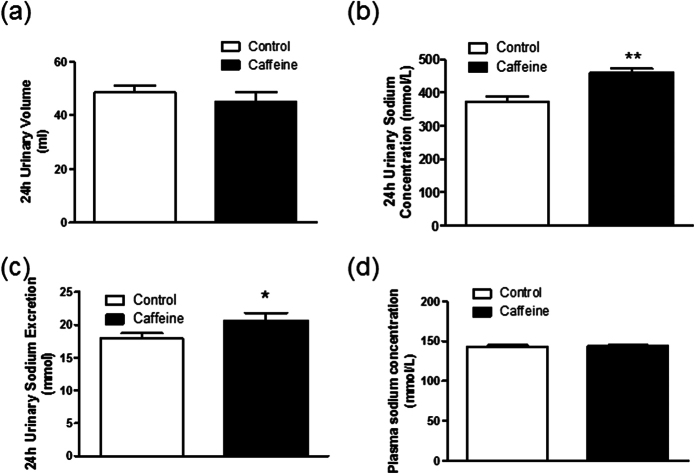
Chronic caffeine intake increases urinary sodium excretion without affecting plasma sodium concentration. (**a**) 24-hour urinary volume was not significantly different after high salt (control) or high salt plus caffeine (caffeine) intervention (n = 5) (**b**) Chronic caffeine intake increased urinary sodium concentration (**c**) Chronic caffeine intake increase 24-hour total urinary sodium excretion (n = 5). (**d**) Plasma sodium concentration analysis showed no difference in control and caffeine group (n = 6). All data are presented as means ± SEM. *P < 0.05, **P < 0.01, compared with control.

To investigate whether chronic caffeine intake has a diuretic or natriuretic effect, we measured the 24-hr urinary volume and urinary sodium concentration of caffeine-treated rats and control rats. Interestingly, despite not altering 24-hr urinary volume (Fig. 3a), chronic caffeine intake increased the urinary sodium concentration and sodium excretion (UNaV) (Fig. 3b,c) without affecting plasma sodium concentration (Fig. 3d). Therefore, increase of urinary sodium excretion would mainly contribute to the anti-hypertensive effect of caffeine.

### Chronic caffeine intake inhibits renal αENaC

**Figure 4:**
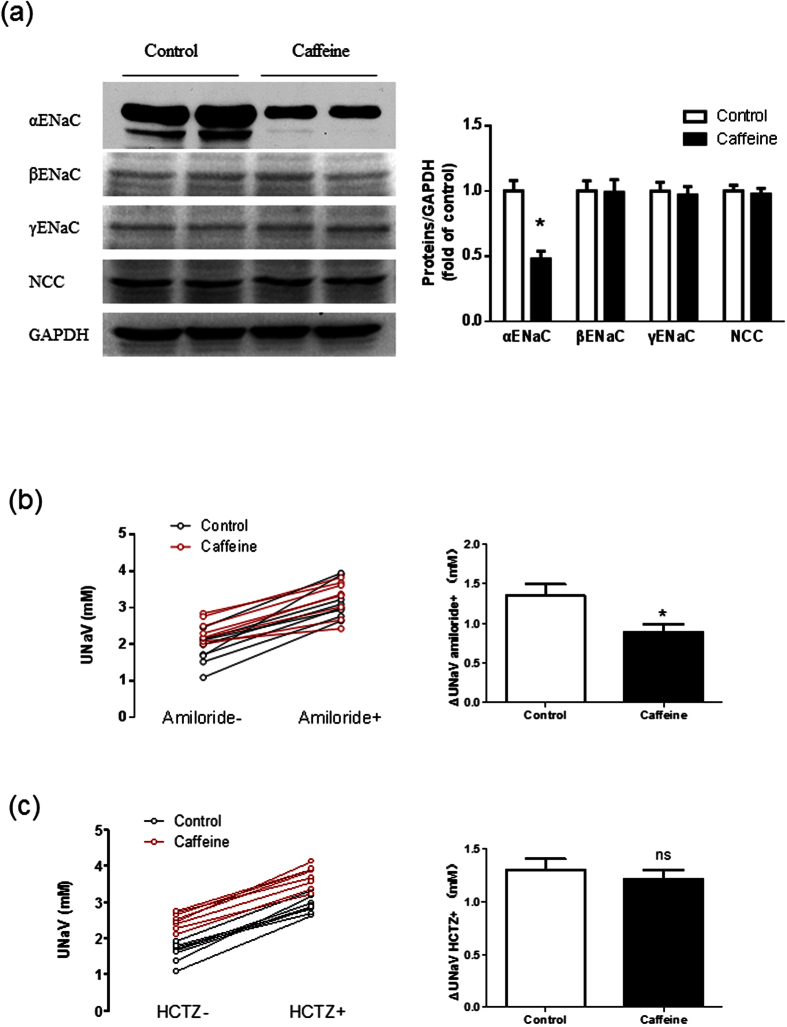
Chronic caffeine intake decreases the expression and function of αENaC in Dahl’s rats. (**a**) Western blot analysis of cortical collecting duct lysates. Chronic caffeine intake deceased αENaC expression without significant influence on βENaC, γENaC and sodium chloride cotransporter (NCC) expression (n = 6). (**b**) Representative curves (left panel) show the individual 24-hour UNaV of rats in control group and caffeine group before and after amiloride injection (3 mg/kg, intraperitoneal). The columns (right panel) show the changes in 24-hour UNaV after amiloride injection (n = 8). (**c**) Representative curves (left panel) show the individual 24-hour UNaV of rats in control group and caffeine group before and after hydrochlorothiazide (HCTZ) injection (3 mg/kg, intraperitoneal). The columns (right panel) show the changes in 24-hour UNaV after HCTZ injection (n = 8). All data are presented as means ± SEM. *P < 0.05 compared with control.

We further examined which renal sodium transporter involved in the effects of caffeine. Long-term administration of caffeine decreased the α-ENaC protein expression in cortical collecting duct of Dahl-S rats, whereas amounts of β- and γ-subunit proteins of ENaC were unchanged. The protein level of the thiazide-sensitive NaCl cotransporter (NCC) in the distal tubule was also unchanged by chronic caffeine intake (Fig. 4a and Supplementary Fig. 1). To establish the importance of ENaC in the effect of caffeine on urinary sodium excretion, Dahl-S rats were given an intraperitoneal injection of amiloride (3 mg/kg), a specific ENaC inhibitor. The ENaC-dependent sodium reabsorption was evaluated by the change in urinary sodium excretion after amiloride intervention. Caffeine-treated Dahl-S rats had a weaker ENaC-dependent sodium reabsorption than controls (Fig. 4b). In contrast, hydrochlorothiazide (12.5 mg/kg) inhibition of renal tubular transport of sodium by NCC was similar in control and caffeine-treated rats (Fig. 4c). Thus, the natriuretic effect of chronic caffeine intake was dependent on renal α-ENaC activity rather than NCC.

### Caffeine treatment attenuates the ENaC function by activating AMPK

**Figure 5:**
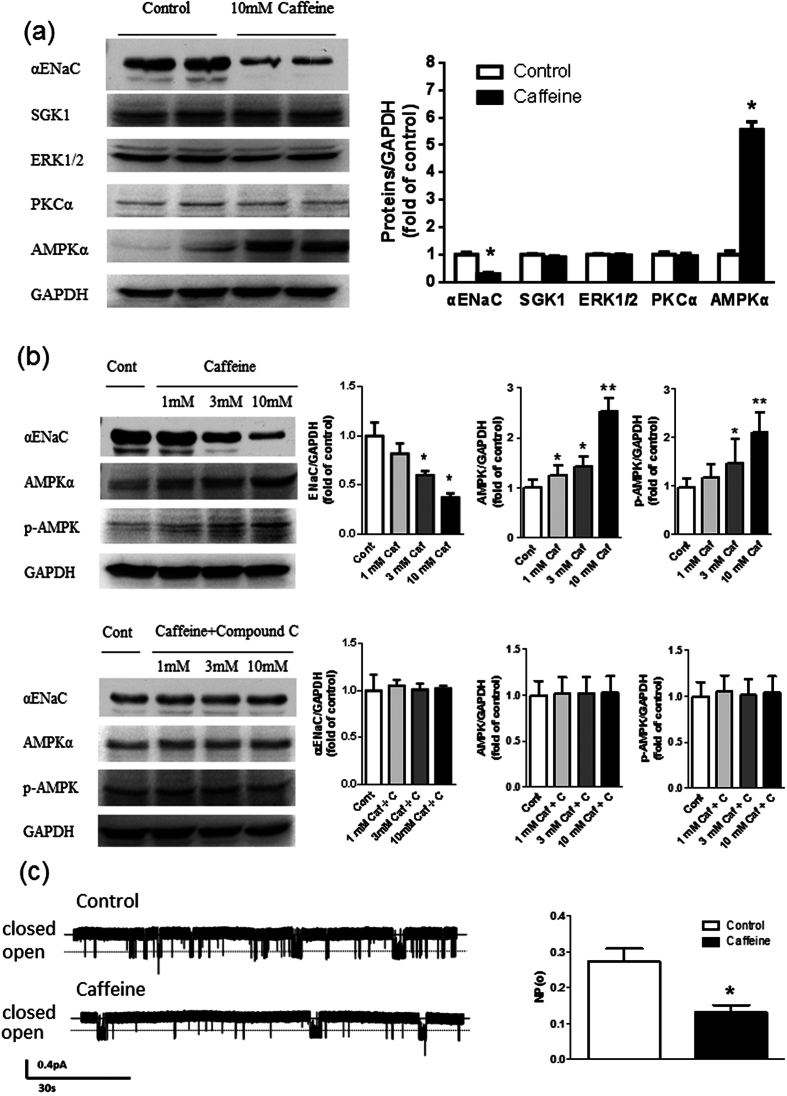
Caffeine decreases the expression and activity of αENaC in M1-CCD cells. (**a**) Western blot analysis showed that incubation with caffeine (10 mM) for 24 hour significantly decreased αENaC expression and increased αAMPK expression without significant impact on SGK1, ERK1/2 and PKCα expression (n = 6). (**b**) Incubation with caffeine (Caf, 1 mM to 10 mM) dose-dependently deceased ENaC expression in M1-CCD cells, but increased AMPK and phosph-AMPK expression in M1-CCD cells. Compound C (C, 1 μM) abolished the effect of caffeine on ENaC, AMPK and phosph-AMPK expression. (n = 5). (**c**) Representative single-channel ENaC traces respectively recorded from two groups of M1-CCD cells by using saline or caffeine to the basolateral bath; downward events represent channel openings; the currents were monitored for at least 30 min. (n = 8). NPo, the product of the number of channels and the open probability was used to measure the channel activity within a patch. All data are presented as means ± SEM. *P < 0.05 compared with control, **P < 0.01 compared with control.

To investigate the mechanism underlying caffeine-induced increase in urinary sodium excretion mediated byα-ENaC inhibition, the expressions of kinase proteins were measured in cortical collecting duct cells (M1-CCD). Caffeine exposure for 24 hr decreased α-ENaC protein expression without affecting protein levels of SGK1, ERK1/2 and PKCα. An exception was αAMPK and its phosphorylated form, which was increased in response to caffeine (Fig. 5a and Supplementary Fig. 2a). Thus we determined whether the effect of caffeine on α-ENaC subunit expression was dependent on AMPK. Caffeine exerted dose-dependent effects to increase αAMPK and phosph-AMPK protein levels in association with reduced α-ENaC. Furthermore, compound C, an AMPK inhibitor, abolished the effects of caffeine on αAMPK, phosph-AMPK and α-ENaC protein levels (Fig. 5b and Supplementary Fig. 2b). In addition, electrophysiological assessment of ENaC activity in M1-CCD cells demonstrated that caffeine exposure for 24 hr decreased the open probability of ENaC (Fig. 5c). These data indicate that caffeine treatment reduces protein levels of the α-ENaC by a mechanism involving AMPK and decreases the open-probability of ENaC in the renal collecting duct that leads to reduced sodium reabsorption and increased sodium excretion.

## Discussion

The present study demonstrates that chronic caffeine administration promotes urinary sodium excretion and reduces blood pressure in salt sensitive hypertensive rats. Furthermore, this effect of caffeine is independent of the sympathetic nerve activation and vascular function. The natriuretic action of caffeine is mediated by inhibition of renal αENaC function without affecting the thiazide-sensitive NaCl cotransporter. In both *in vitro* and *in vivo* studies, caffeine up-regulated αAMPK level which suppressed αENaC expression and activity.

Caffeine is a major ingredient in a number of the most widely consumed non-alcoholic beverages^27^. Although the diuretic and natriuretic effects of caffeine have been recognized for long term, the mechanism responsible for caffeine effect was not fully elucidated. Thomsen *et al*. reported that acute caffeine intake increases lithium clearance that reflects sodium reabsorption in proximal tubule^28^. Rieg *et al*. showed that the acute diuresis and natriuresis produced by caffeine were related to blockade of adenosine A_1_ receptors^29^. However, the chronic effects of caffeine on renal sodium handling are rarely investigated. The diuretic effect of caffeine is short-lived and is easy to develop tolerance^30^. In addition, no dehydration was observed in habitual coffee drinkers^31^. These findings suggest that other mechanism might be involved when chronic caffeine use. In the present study, we found that chronic caffeine intake dominantly increased 24-hr urinary sodium excretion but not urinary volume in Dahl-S rats compared with control rats. This natriuretic effect but not diuretic effect suggested a primary action on renal sodium handling in chronic caffeine intake.

In our study, we showed that the urinary sodium excretion increased after caffeine intervention without change in plasma sodium concentration. Similarly, Burge *et al*. reported that long-term diuretics (amiloride and hydrochlorothiazide) administration at therapeutic dose have no influence on plasma sodium concentration^32^. Since caffeine displays a much weaker natriuretic effect than diuretics^9^, chronic caffeine administration would not affect plasma osmolality.

It is well documented that thiazide-sensitive NaCl cotransporter (NCC), amiloride-sensitive epithelial sodium channel (ENaC), and sodium hydrogen (Na/H) exchanger are major regulators of renal tubular sodium reabsorption thus affect blood pressure regulation^18^,^19^. These epithelial sodium transporters can be regulated by several kinases such as protein kinase C, with-no-lysine kinase (WNK) 4 and 1, ERK 1/2, and serum- and glucocorticoid-inducible protein kinase 1 (SGK1), as well as AMPK^16^,^21^. Disturbances of signaling via these kinase pathways can result in human sodium retention and hypertension^33^,^34^,^35^,^36^. Previous studies in oocytes and epithelial tissues showed that AMPK inhibits sodium transport through increasing Nedd4-2-dependent ENaC retrieval from the membrane^37^,^38^. Some studies reported that caffeine can activate AMPK in different type of cells^22^,^23^,^24^. In the *in vivo* study, chronic caffeine intake decreased the protein levels and function of ENaC, but not NCC, which also affects renal electrolyte transport and blood pressure in the distal convoluted tubule^39^. Thus, caffeine appears to increase urinary sodium excretion by inhibiting renal ENaC activity secondary to the AMPK pathway. However, whether caffeine inhibits the open probability of ENaC by regulating its expression or ability still needs further investigation.

We also confirmed that acute caffeine intake induced an initial increase in blood pressure in association with a transient increase in locomotor activity and heart rate in Dahl-S rats, which is likely associated with sympathetic nerve activation. By contrast, these effects failed to be observed in long-term caffeine intake. Importantly, long-term administration of caffeine lowered blood pressure, which was not associated with sympathetic nerve activation and cardiovascular changes in Dahl-S rats. It suggested that chronic hypotensive effects of caffeine could be caused by its natriuretic effects in Dahl-S rats. Some studies have reported that systolic blood pressure, rather than diastolic blood pressure, are affected by urinary sodium excretion^40^,^41^. This may partially explain why caffeine failed to prevent high salt induced increase in diastolic blood pressure in our study. In addition, a recent study also showed that ambulatory systolic blood pressure was inversely correlated with urinary caffeine and its metabolites in adults from a general population, which indicated a potential protective effect of caffeine on blood pressure^42^. Thus, chronic caffeine intake might be an effective lifestyle intervention to promote salt excretion in people who normally consume a high salt diet. However, its application in human still warrants further determination in future.

In conclusion, we have demonstrated that chronic caffeine intake prevents salt-sensitive hypertension in Dahl-S rats. The beneficial effect of caffeine is associated with activation of renal AMPK that inhibits ENaC activity, which subsequently increases urinary sodium excretion and maintains blood pressure during high salt diet. However, chronic effect of caffeine on animal models needs to be further validated in human. These findings provide insight into the physiological role of caffeine in a long-term regulation of blood pressure through affecting renal sodium handling.

## Materials and Methods

### Animal Treatment

Six-week-old male Dahl salt-sensitive rats (Dahl-S) were obtained from Vital River Company, Beijing, China and housed under controlled temperature (21–23 °C) with a 12/12 hr light-dark cycle with free access to food and water. Animals were anesthetized by inhaling of 2% isoflurane (v/v), and surgically implanted with BP telemetric transmitters (TA11PA-C40, Data Sciences International, Minnesota, USA). After recovering from the surgery for 10 days, animals were randomly assigned to two groups: The control group was given a high salt chow containing 8% (w/w) NaCl and normal drinking water. The caffeine group was fed the 8% NaCl chow and 0.1% caffeine in their drinking water. The dietary intervention lasted 15 days. All of the experimental procedures were performed in accordance with protocols approved by Institutional Animal Care and Use Committee of the Institute of Third Military Medical University, and all experiments were performed in accordance with the National Institutes of Health guidelines for the use of experimental animals.

### Blood pressure and locomotor activity measurement

24-hour ambulatory blood pressures and locomotor activity were measured by radiotelemetry in conscious, unrestrained rats as previously described^43^. We collected data for 10 seconds every 30 minutes and used the mean values of 24 hours for the analysis.

### Urinary Samples Analysis

On the 16th day, rats were transferred to individual metabolic cages (Tecniplast, Italy). Both groups maintained the same dietary intervention as mentioned before and had free access to food and water. The 24-hr water consumption and urinary excretion of water and sodium were measured. Urinary sodium concentrations were measured using a flame photometer (Spectrum, Shanghai China). Urinary sodium excretion (UNaV) was calculated by this formula: UNaV = [urine sodium concentration] × [24-hour urine volume].

### Vascular Reactivity and Electrical Field Stimulation

Vascular reactivity and electrical field stimulation were performed as previously described^44^. After rats were anesthetized with pentobarbital sodium (100 mg/kg body weight ip), the mesenteric vascular bed was removed and placed in a cold (4 °C) Krebs solution containing (mM): 118 NaCl, 25 NaHCO_3_, 11 D-glucose, 4.7 KCl, 1.2 KH_2_PO_4_, 1.17 MgSO_4_, and 2.5 CaCl_2_. The second branches of mesenteric arteries were dissected out and the connective tissue was removed. The arterial segments (2–2.5 mm in length) were mounted in a myograph. Vascular rings were bathed in Krebs solution aerated with 95% O_2_ and 5% CO_2_ at 37 °C (pH 7.4) and were stretched to the optimum baseline tension (2.5 mN). The rings were equilibrated for 60 min before the start of an experiment. High K^+^ (60 mM)-containing Krebs solution was added to test contractility. Isometric contractions were recorded using a computerized data acquisition system (PowerLab/8SP; AD Instruments Pty Ltd., Castle Hill, Australia).

Electrical Field Stimulation (EFS) was achieved using a stimulator (Grass SD9) connected to two platinum electrodes placed on each side of the ring parallel to its longitudinal axis. The frequency–response curves (2–32 Hz, 30 V, 30 s trains and 1 ms duration) were obtained. There was an interval of 1 min between each stimulus to allow for recovery of basal tone. To evaluate the neural origin of the EFS-induced contractile response, the nerve impulse propagation blocker, tetrodotoxin (TTX, 0.1 mM), was added to the bath 30 min before the second frequency-response curve was determined.

### Preparation of renal cortical collecting duct

Rats were anesthetized with sodium pentobarbital (100 mg/kg body weight ip), the circulatory system was perfused via the left ventricle with Eagle’s minimal essential medium (MEM) containing collagenase (1 mg/ml), soybean trypsin inhibitor (2 μg/ml), aprotinin (2 μg/ml). After perfusion, kidneys were removed, cut into coronal slices, and incubated for 10–15 min at 37 °C in the same solution used for the perfusion. After collagenase washout, slices were kept in ice-cold MEM containing soybean trypsin inhibitor (2 μg/ml) throughout the microdissection procedure. The renal cortical collecting duct (CCD) segments were separated manually using fine forceps and identified with characteristic branching indicative of CCDs. The pools of CCDs containing 10–20 microdissected tubules with the total tubular length of ~10 mm/pool were transferred in 5 μl of Dulbecco’s modified Eagle’s medium (DMEM)/ Ham’s F-12 (1:1) medium into 1.5 ml of Eppendorf tubes and refrigerated in −70 °C for protein extraction^45^.

### The amiloride and hydrochlorothiazide test

Another group of rats were used to evaluate the effect of caffeine on ENaC and NCC function at distal nephron. The procedure of fifteen-day’s dietary intervention was the same as mentioned at Animal Treatment section. After that rats were transferred to metabolic cages. A 24-hr urine samples were collected in metabolic cages. Then the rats were administrated of amiloride (3 mg/kg, ip), an ENaC antagonist, or hydrochlorothiazide (12.5 mg/kg, ip), a NCC antagonist. After the amiloride or hydrochlorothiazide administration, another 24-hr urine samples were collected. Urinary sodium concentrations were measured using a flame photometer (Spectrum, Shanghai China).

### M1-CCD Cells Culture

The renal cortical collecting duct M1-CCD cell line was purchased from American Type Culture Collection (CRL-2038; American Type Culture Collection, Manassas, VA). M1-CCD cells were cultured in DMEM/F-12 (1:1) medium with FBS (5%) supplemented with L-glutamine (2 mM), penicillin (100 mg/ml), streptomycin (100 U/ml), and dexamethasone (5 mM) and grown at 37 °C in a 5% CO_2_ humidified incubator. Quiescent cells were obtained by serum free medium incubation for 12 hr before treatment of caffeine (Sigma, series number: 93784) and the AMPK inhibitor Compound C (Calbiochem, series number: 171260).

### Western Blotting analysis

Western blotting analysis was performed using standard procedures as previously described^46^. The antibodies against αENaC (sc-21012), βENaC (sc-21013), γENaC (sc-21014), SGK1 (sc-28338), ERK1/2 (sc-135960), PKCα (sc-8393), AMPKα (sc-25792) and phosphor-AMPKα (sc-33524) were purchased from Santa Cruz Biotechnology (Santa Cruz, CA, USA). The NCC antibody (AB3553) was a product of Millipore (Merck Millipore Corporation, Darmstadt, Germany) and the internal control GAPDH antibody (KC-5G5) was purchased from Kang Chen Bio-tech Incorporation, China. Transferred PVDF membranes were incubated with primary antibodies at 4 °C for 12 hr. After incubation with secondary antibodies (ZSGB-BIO, China) at room temperature for 2 hr, the proteins were detected with enhanced chemiluminescence and quantified using a Gel Doc 2000 Imager (Bio-Rad, USA). The protein levels were normalized to the internal control GAPDH.

### Electrophysiology

For single channel recordings of ENaC, M1-CCD cells were studied under voltage-clamp conditions using standard methods as previously described. A patch pipette with the resistance of 6–8 MΩ was fabricated from a borosilicate glass capillary (1.5 mm od, 0.86 mm id, Sutter Instrument, Germany) on a Sutter Puller (P97, Sutter Instrument, Germany). The bath solution was (in mM): 110 NaCl, 4.5 KCl, 1 MgCl_2_, 1 CaCl_2_, 5 Hepes, 5 Na-Hepes (pH 7.2) wih a pipette solution of: 110 NaCl, 4.5 KCl, 0.1 EGTA, 5 Hepes, 5 Na-Hepes (pH 7.2). Single-channel currents were recorded using an EPC-10 patch-clamp amplifier (HEKA Instrument, Germany) and PATCHMASTER 8.0 software (HEKA Instruments, Germany). The data were acquired by application of 0 mV pipette potential and were sampled at 5 KHz and low-pass filtered at 1 KHz. During *post hoc* analysis, data were further filtered at 50 Hz and single-channel events were listed and analyzed by clamp fit 10.2 software (Molecular Devices, Sunnyvale, CA, USA). The total number of functional channels in a patch was determined by observing the number of peaks detected on the current amplitude histograms during at least 10-min recording period. NPo, the product of the number of channels and the open probability, or the open probability (Po) of ENaC before and after application of test agents was calculated using Clampfit 10.2 (Molecular Devices, Sunnyvale, CA, USA). In single-channel records, the control ENaC activity was recorded for 3–4 min after forming the cell-attached mode and ENaC activity had stabilized. We usually recorded at least 30 min of any experimental manipulation.

### Statistical Analysis

All data are expressed as mean ± SEM. Statistical significant differences between two groups were evaluated by means of Mann-Whitney U tests. P values < 0.05 were considered to be statistically significant.

## Additional Information

**How to cite this article**: Yu, H. *et al*. Caffeine intake antagonizes salt sensitive hypertension through improvement of renal sodium handling. *Sci. Rep*. **6**, 25746; doi: 10.1038/srep25746 (2016).

## Supplementary Material

Supplementary Information

## References

[b1] Bibbins-Domingo K. . Projected effect of dietary salt reductions on future cardiovascular disease. N Engl J Med. 362, 590–599 (2010). 20089957 10.1056/NEJMoa0907355 PMC3066566

[b2] Ibrahim M. M. & Damasceno A. Hypertension in developing countries. Lancet 380, 611–619 (2012). 22883510 10.1016/S0140-6736(12)60861-7

[b3] Haddy F. J. Role of dietary salt in hypertension. Life Sci 79, 1585–1592 (2006). 16828490 10.1016/j.lfs.2006.05.017

[b4] He F. J. & MacGregor G. A. Salt reduction lowers cardiovascular risk: meta-analysis of outcome trials. Lancet 378, 380–382 (2011). 21803192 10.1016/S0140-6736(11)61174-4

[b5] Powles J. . Global, regional and national sodium intakes in 1990 and 2010: a systematic analysis of 24 h urinary sodium excretion and dietary surveys worldwide. BMJ Open 3, e003733 (2013). 10.1136/bmjopen-2013-003733 PMC3884590 24366578

[b6] Hanon O. . Diuretics for cardiovascular prevention in the elderly. J Hum Hypertens 18, Suppl 2, S15–22 (2004). 15592568 10.1038/sj.jhh.1001796

[b7] Smith A. Effects of caffeine on human behavior. Food Chem Toxicol 40, 1243–1255 (2002). 12204388 10.1016/s0278-6915(02)00096-0

[b8] Ogawa N. & Ueki H. Clinical importance of caffeine dependence and abuse. Psychiatry Clin Neurosci 61, 263–268 (2007). 17472594 10.1111/j.1440-1819.2007.01652.x

[b9] Osswald H. & Schnermann J. Methylxanthines and the kidney. Handb Exp Pharmacol 200, 391–412 (2011). 20859805 10.1007/978-3-642-13443-2_15 PMC3275788

[b10] Myers M. G. Effect of caffeine on blood pressure beyond the laboratory. Hypertension 43, 724–725 (2004). 14967826 10.1161/01.HYP.0000120970.49340.33

[b11] Umemura T. . Effects of acute administration of caffeine on vascular function. Am J Cardiol 98, 1538–1541 (2006). 17126666 10.1016/j.amjcard.2006.06.058

[b12] Conde S. V. . Chronic caffeine intake decreases circulating catecholamines and prevents diet-induced insulin resistance and hypertension in rats. Br J Nutr 107, 86–95 (2012). 21733336 10.1017/S0007114511002406

[b13] Guarino M. P., Ribeiro M. J., Sacramento J. F. & Conde S. V. Chronic caffeine intake reverses age-induced insulin resistance in the rat: effect on skeletal muscle Glut4 transporters and AMPK activity. *Age* (*Dordr*) 35, 1755–1765 (2013). 22976123 10.1007/s11357-012-9475-x PMC3776116

[b14] Panchal S. K., Wong W. Y., Kauter K., Ward L. C. & Brown L. Caffeine attenuates metabolic syndrome in diet-induced obese rats. Nutrition 28, 1055–1062 (2012). 22721876 10.1016/j.nut.2012.02.013

[b15] Yeh T. C. . Caffeine intake improves fructose-induced hypertension and insulin resistance by enhancing central insulin signaling. Hypertension 63, 535–541 (2014). 24366086 10.1161/HYPERTENSIONAHA.113.02272

[b16] Vallon V. Regulation of the Na^+^-Cl^−^ cotransporter by dietary NaCl: a role for WNKs, SPAK, OSR1, and aldosterone. Kidney Int 74, 1373–1375 (2008). 19008908 10.1038/ki.2008.477 PMC2944250

[b17] Coffman T. M. Under pressure: the search for the essential mechanisms of hypertension. Nat Med 17, 1402–1409 (2011). 22064430 10.1038/nm.2541

[b18] Eladari D., Chambrey R., Picard N. & Hadchouel J. Electroneutral absorption of NaCl by the aldosterone-sensitive distal nephron: implication for normal electrolytes homeostasis and blood pressure regulation. Cell Mol Life Sci 71, 2879–2895 (2014). 24556999 10.1007/s00018-014-1585-4 PMC11113337

[b19] Wang X., Armando I., Upadhyay K., Pascua A. & Jose P. A. The regulation of proximal tubular salt transport in hypertension: an update. Curr Opin Nephrol Hypertens 18, 412–420 (2009). 19654544 10.1097/MNH.0b013e32832f5775 PMC3722593

[b20] Thomas C. P. & Itani O. A. New insights into epithelial sodium channel function in the kidney: site of action, regulation by ubiquitin ligases, serum- and glucocorticoid-inducible kinase and proteolysis. Curr Opin Nephrol Hypertens 13, 541–548 (2004). 15300161 10.1097/00041552-200409000-00010

[b21] Baines D. Kinases as targets for ENaC regulation. Curr Mol Pharmacol 6, 50–64 (2013). 23547935 10.2174/18744672112059990028

[b22] Egawa T. . Caffeine acutely activates 5′adenosine monophosphate-activated protein kinase and increases insulin-independent glucose transport in rat skeletal muscles. Metabolism 58, 1609–1617 (2009). 19608206 10.1016/j.metabol.2009.05.013

[b23] Quan H. Y., Kim do Y. & Chung S. H. Caffeine attenuates lipid accumulation via activation of AMP-activated protein kinase signaling pathway in HepG2 cells. BMB Rep 46, 207–212 (2013). 23615262 10.5483/BMBRep.2013.46.4.153 PMC4133884

[b24] Spyridopoulos I. . Caffeine enhances endothelial repair by an AMPK-dependent mechanism. Arterioscler Thromb Vasc Biol 28, 1967–1974 (2008). 18757291 10.1161/ATVBAHA.108.174060

[b25] Rapp J. P. Dahl salt-susceptible and salt-resistant rats. A review. Hypertension 4, 753–763 (1982). 6754600 10.1161/01.hyp.4.6.753

[b26] Zicha J. . Age-dependent salt hypertension in Dahl rats: fifty years of research. Physiol Res 61, Suppl 1, S35–87 (2012). 22827876 10.33549/physiolres.932363

[b27] Heckman M. A., Weil J. & Gonzalez de Mejia E. Caffeine (1, 3, 7-trimethylxanthine) in foods: a comprehensive review on consumption, functionality, safety, and regulatory matters. J Food Sci 75, R77–87 (2010). 20492310 10.1111/j.1750-3841.2010.01561.x

[b28] Thomsen K. & Schou M. Renal lithium excretion in man. Am J Physiol 215, 823–827 (1968). 5676383 10.1152/ajplegacy.1968.215.4.823

[b29] Rieg T. . Requirement of intact adenosine A1 receptors for the diuretic and natriuretic action of the methylxanthines theophylline and caffeine. J Pharmacol Exp Ther 313, 403–409 (2005). 15590766 10.1124/jpet.104.080432

[b30] Maughan R. J. & Griffin J. Caffeine ingestion and fluid balance: a review. J Hum Nutr Diet 16, 411–420 (2003). 19774754 10.1046/j.1365-277x.2003.00477.x

[b31] Killer S. C., Blannin A. K. & Jeukendrup A. E. No evidence of dehydration with moderate daily coffee intake: a counterbalanced cross-over study in a free-living population. Plos One 9, e84154 (2014). 24416202 10.1371/journal.pone.0084154 PMC3886980

[b32] Burge P. S. & Montuschi E. Long-term effects of an amiloride/hydrochlorothiazide combination (‘Moduretic’) on electrolyte balance. Curr Med Res Opin 4, 260–266 (1976). 991618 10.1185/03007997609109315

[b33] Farjah M., Roxas B. P., Geenen D. L. & Danziger R. S. Dietary salt regulates renal SGK1 abundance: relevance to salt sensitivity in the Dahl rat. Hypertension 41, 874–878 (2003). 12642512 10.1161/01.HYP.0000063885.48344.EA

[b34] Lalioti M. D. . Wnk4 controls blood pressure and potassium homeostasis via regulation of mass and activity of the distal convoluted tubule. Nat Genet 38, 1124–1132 (2006). 16964266 10.1038/ng1877

[b35] Davies M. . Novel mechanisms of Na^+^ retention in obesity: phosphorylation of NKCC2 and regulation of SPAK/OSR1 by AMPK. Am J Physiol Renal Physiol 307, F96–F106 (2014). 24808538 10.1152/ajprenal.00524.2013

[b36] Deji N. . Role of angiotensin II-mediated AMPK inactivation on obesity-related salt-sensitive hypertension. Biochem Biophys Res Commun 418, 559–564 (2012). 22293193 10.1016/j.bbrc.2012.01.070

[b37] Carattino M. D. . Epithelial sodium channel inhibition by AMP-activated protein kinase in oocytes and polarized renal epithelial cells. J Biol Chem 280, 17608–17616 (2005). 15753079 10.1074/jbc.M501770200

[b38] Bhalla V. . AMP-activated kinase inhibits the epithelial Na+ channel through functional regulation of the ubiquitin ligase Nedd4-2. J Biol Chem 281, 26159–26169 (2006). 16844684 10.1074/jbc.M606045200

[b39] Dimke H. Exploring the intricate regulatory network controlling the thiazide-sensitive NaCl cotransporter (NCC). Pflugers Arch 462, 767–777 (2011). 21927811 10.1007/s00424-011-1027-1 PMC3215886

[b40] Cugini P. . Usefulness of twenty-four-hour blood pressure patterns and response to short-term sodium restriction in normotensive subjects in detecting a predisposition to systemic arterial hypertension. Am J Cardiol 64, 604–608 (1989). 2782250 10.1016/0002-9149(89)90487-6

[b41] Harshfield G. A. . Sodium excretion and racial differences in ambulatory blood pressure patterns. Hypertension 18, 813–818 (1991). 1743762 10.1161/01.hyp.18.6.813

[b42] Guessous I. . Associations of ambulatory blood pressure with urinary caffeine and caffeine metabolite excretions. Hypertension 65, 691–696 (2015). 25489060 10.1161/HYPERTENSIONAHA.114.04512

[b43] Sun J. . Activation of cold-sensing transient receptor potential melastatin subtype 8 antagonizes vasoconstriction and hypertension through attenuating RhoA/Rho kinase pathway. Hypertension 63, 1354–1363 (2014). 24637663 10.1161/HYPERTENSIONAHA.113.02573

[b44] Zhang H. . Gastrointestinal intervention ameliorates high blood pressure through antagonizing overdrive of the sympathetic nerve in hypertensive patients and rats. J Am Heart Assoc 3, e000929 (2014). 25240055 10.1161/JAHA.114.000929 PMC4323786

[b45] Nesterov V., Dahlmann A., Bertog M. & Korbmacher C. Trypsin can activate the epithelial sodium channel (ENaC) in microdissected mouse distal nephron. Am J Physiol Renal Physiol 295, F1052–1062 (2008). 18653483 10.1152/ajprenal.00031.2008

[b46] Ma S. . Activation of the cold-sensing TRPM8 channel triggers UCP1-dependent thermogenesis and prevents obesity. J Mol Cell Biol 4, 88–96 (2012). 22241835 10.1093/jmcb/mjs001

