# Supplementary material for: Caffeine intake antagonizes salt sensitive hypertension through improvement of renal sodium handling
Source: Sci Rep. 2016 May 12;6:25746. doi: 10.1038/srep25746 (PMC4866033; doi:10.1038/srep25746)

Caffeine intake antagonizes salt sensitive hypertension through improvement of renal sodium handling

Supplementary Information

Hao Yu#, Tao Yang#, Peng Gao, Xing Wei, Hexuan Zhang, Shiqiang Xiong, Zongshi Lu, Li Li, Xiao Wei, Jing Chen, Yu Zhao, William J. Arendshorst, Qianhui Shang*, Daoyan Liu, Zhiming Zhu*

#Authorship Note: Hao Yu and Tao Yang contributed equally to this work.

*Corresponding author: Zhiming Zhu and Qianhui Shang,
E-mail: zhuzm@yahoo.com or qhshang60@126.com
Phone numbers: +86-23-68767849 Fax numbers: +86-23-68705094

Supplementary Figure 1.

Original Western Blot figures in Figure 4a. Black arrowhead indicates the target band of antibody which is presented in manuscript.

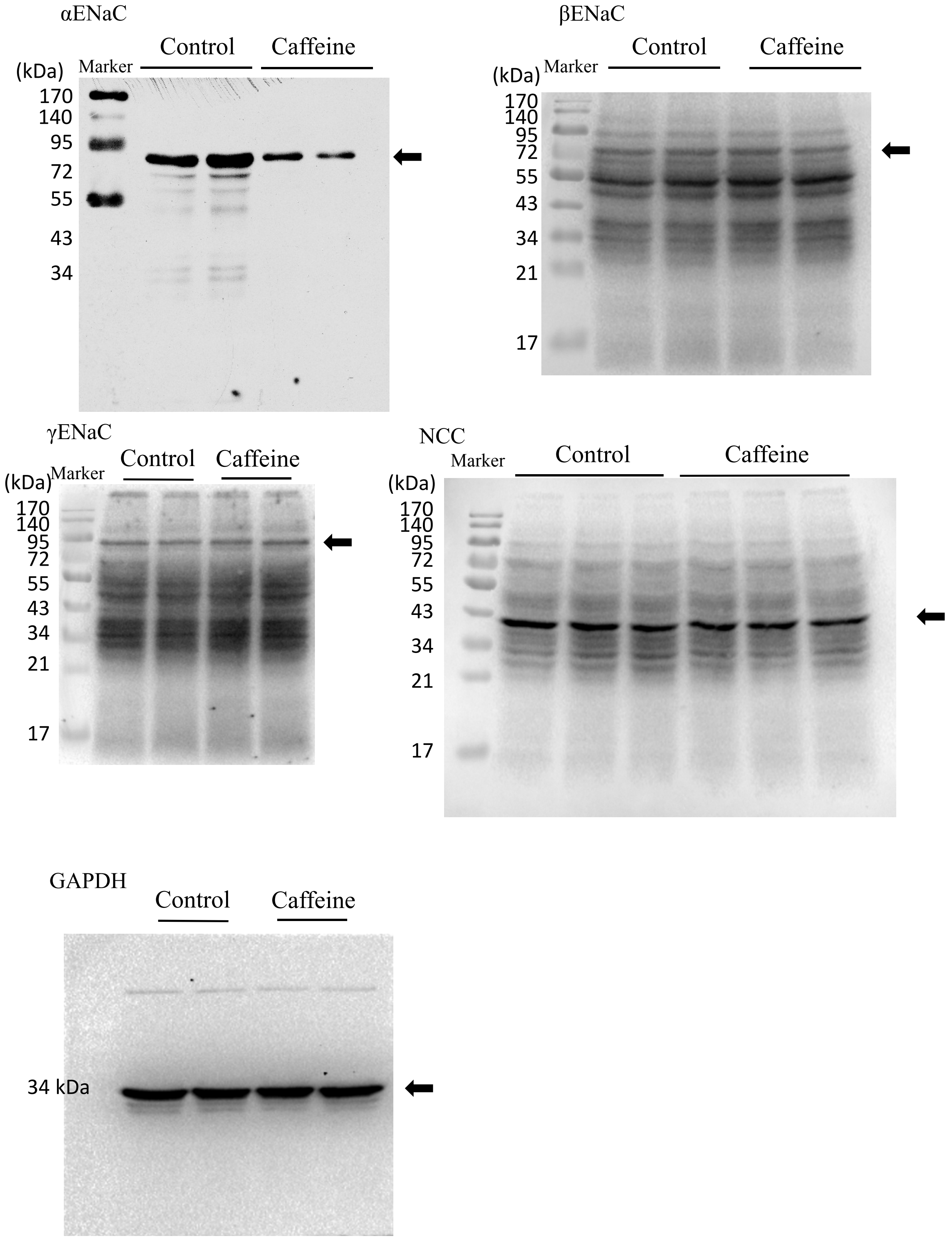

Supplementary Figure 2

Original Western Blot figures in Figures 5a and 5b. Black arrowhead indicates the target band of antibody which is presented in manuscript.

(a)

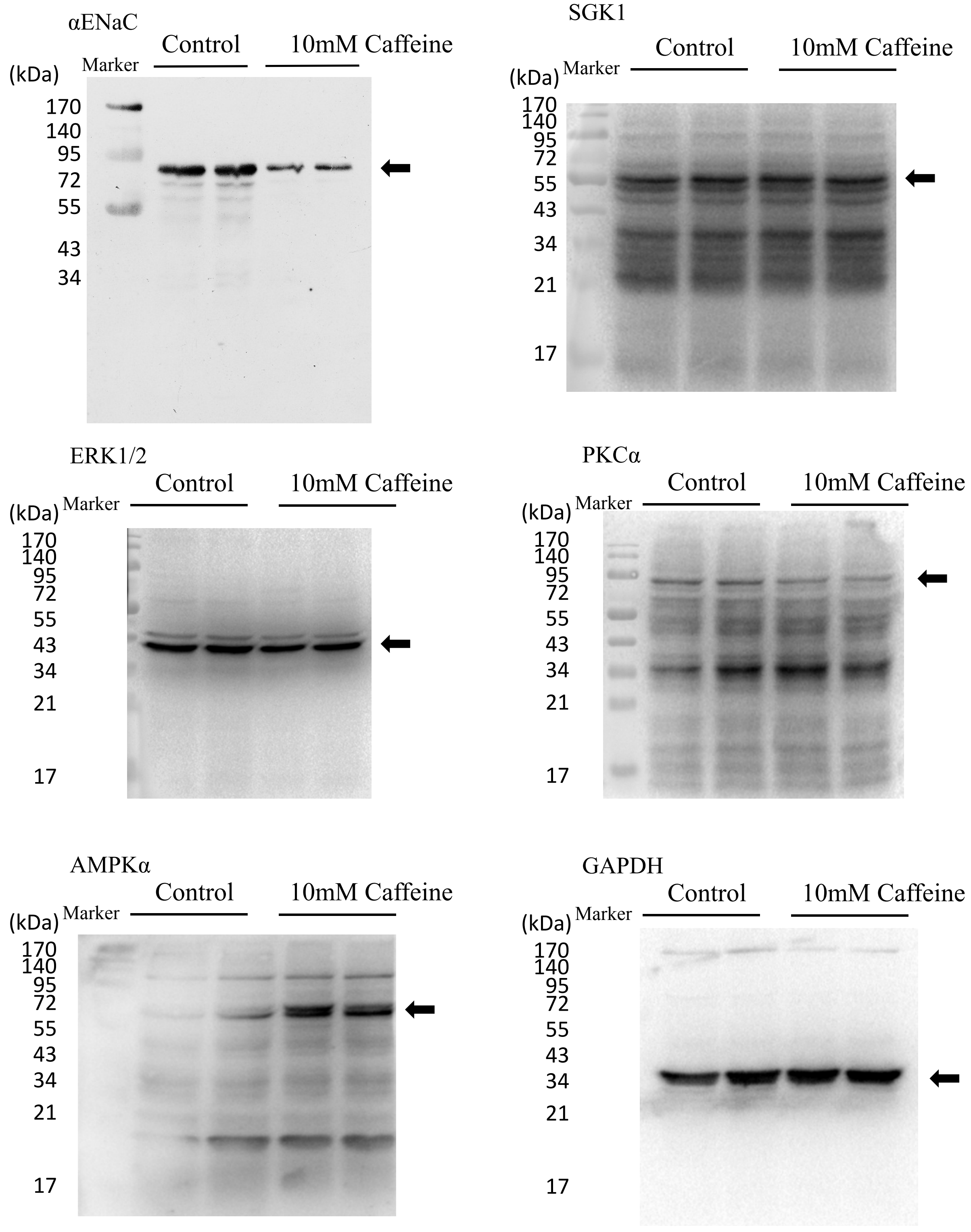

Supplemental Figure 2

(b)

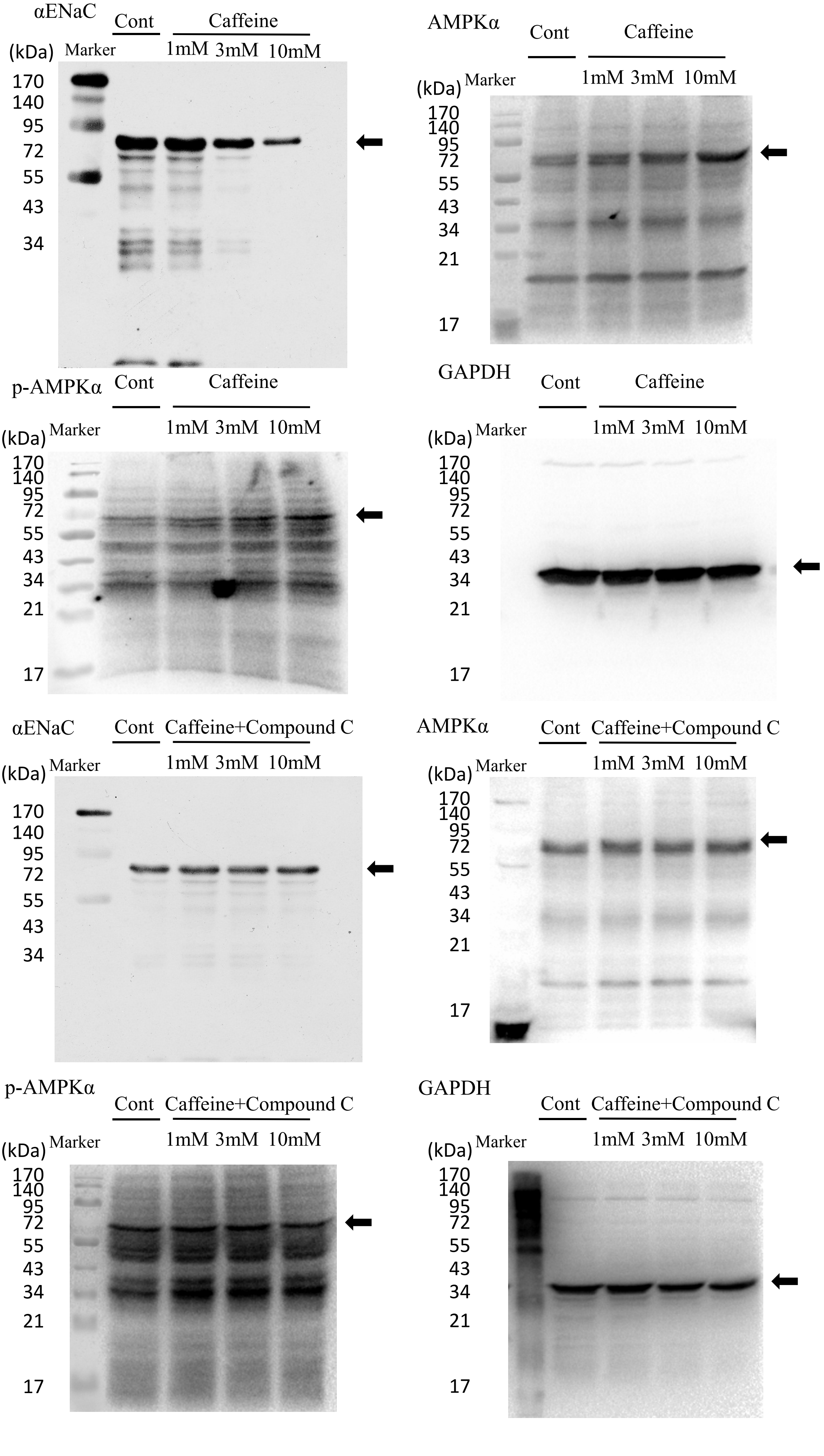

Supplement: Supplementary Information [file srep25746-s1.pdf]
